# Clinical Evidence of the Use of Mepolizumab in the Treatment of Chronic Rhinosinusitis with Nasal Polyps: A Prospective Observational Study

**DOI:** 10.3390/healthcare13040419

**Published:** 2025-02-15

**Authors:** Antonio Moffa, Francesco Iafrati, Lucrezia Giorgi, Domiziana Nardelli, Luca Carnuccio, Peter Baptista, Ewa Olszewska, Manuele Casale

**Affiliations:** 1 School of Medicine, Università Campus Bio-Medico di Roma, 00128 Rome, Italy; francesco.iafrati@policlinicocampus.it (F.I.); domiziana.nardelli@alcampus.it (D.N.); luca.carnuccio@unicampus.it (L.C.); m.casale@policlinicocampus.it (M.C.); 2 Integrated Therapies in Otolaryngology, Fondazione Policlinico Universitario Campus Bio-Medico, 00128 Rome, Italy; l.giorgi@unicampus.it; 3 ENT Department, Al Zahra Private Hospital Dubai, Dubai 23614, United Arab Emirates; peterbaptista@rocketmail.com; 4 Department of Otolaryngology, Sleep Apnea Surgery Center, Medical University of Bialystok, 15-276 Bialystok, Poland; ewa.olszewska@umb.edu.pl

**Keywords:** MFepolizumab, chronic rhinosinusitis, nasal polyps

## Abstract

**Background/Objectives**: Chronic rhinosinusitis with nasal polyps (CRSwNP) poses significant therapeutic challenges. The introduction of Mepolizumab, an anti-interleukin-5 monoclonal antibody, offers a new therapeutic option for patients with severe, uncontrolled CRSwNP. This prospective observational study aims to assess the efficacy and safety of Mepolizumab for treating severe CRSwNP in Italy. **Methods**: A single-center prospective observational study conducted in real-life settings with the patients of our center. Prior to enrollment, each patient underwent an interdisciplinary evaluation involving a pulmonologist and an allergologist if deemed necessary. All patients who were referred for treatment with Mepolizumab in compliance with the AIFA guidelines and the EPOS/EUFOREA update were included in the study population: (1) subjects who were over the age of 18, (2) who had severe CRSwNP, (3) whose condition was not successfully managed with standard therapies alone, and (4) whose blood eosinophil counts were greater than 150 cells/mL. Mepolizumab was administered subcutaneously through a 100 mg injection once every four weeks in addition to the standard-of-care therapy. **Results**: At the end of the enrollment process, 20 patients with severe CRSwNP were enrolled. Significant improvements were observed in Nasal Polyp Score, quality of life (SNOT-22; *p* < 0.05), and nasal obstruction and rhinorrhea (*p* < 0.05), while no significant improvements were seen in olfactory function (*p* < 0.05). Eosinophil levels also significantly decreased (*p* < 0.05). **Conclusions**: Mepolizumab effectively manages severe CRSwNP, showing improvements in symptom control and quality of life with an acceptable safety profile.

## 1. Introduction

**Figure 1:**
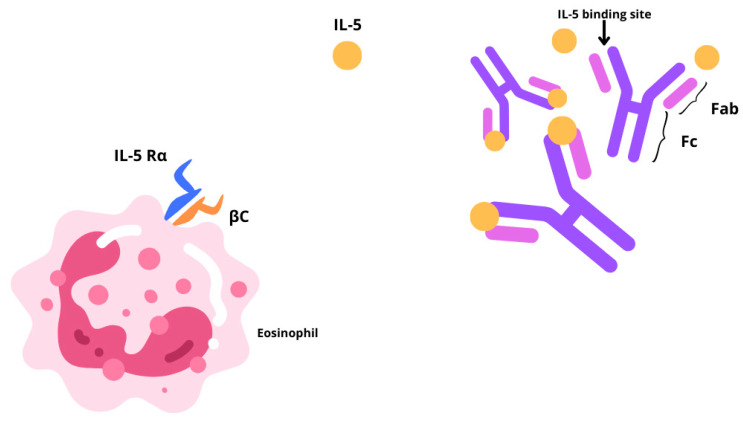
Graphic representation illustrating the action of Mepolizumab through a IL-5 blockade, which hinders its association with the IL-5 receptor alpha (IL-5Rα).

Chronic rhinosinusitis (CRS) is a multifactorial inflammatory disease with two main different phenotypes, CRS without nasal polyps (CRSsNP) or with nasal polyps (CRSwNP) [1,2]. CRSsNP, characterized by fibrosis, basement membrane thickening, and goblet cell hyperplasia, affects around 80% of CRS cases. The remaining 20% of CRS patients show a bilateral development of benign oedematous nasal polyps, from the ethmoid sinuses to the nasal cavities. The prevalence of CRSwNP in the global population is estimated to be 1–4%, although epidemiological data are limited [3,4]. Often, CRSwNP is found in association with other respiratory conditions including asthma, idiopathic bronchiectasis, or aspirin sensitivity. About 20–60% of patients affected by CRSwNP reportedly display comorbid asthma [5]. CRSwNP is predominantly characterized by a type 2 inflammatory phenotype, associated with a type 2 (T2) cellular response and epithelial barrier, and mucociliary, and olfactory dysfunction. In particular, the interleukins (IL)-4, IL-13, and IL-5 are pivotal drivers and perpetrators of type 2 inflammation [6]. In these settings, cytokine-driven structural changes include nasal epithelial tissue remodeling, and nasal polyp formation, and together contribute to sustained barrier dysfunction. Type 2 inflammation may also hinder the olfactory sensory neurons in a quantitative or qualitative manner, thereby contributing to anosmia or hyposmia. CRSwNP has a high clinical burden. It is characterized by persistent, bothersome symptoms [1], some of which lead to sleep disturbance [7], reducing patient concentration and work productivity [8]. One of the most bothersome symptoms for CRSwNP patients is olfactory dysfunction, specifically the loss of smell and taste, which greatly impacts health-related quality of life [9]. The standard-of-care treatment for CRSwNP entails Intranasal Corticosteroids (INCS) together with short courses of Oral Corticosteroids (OCSs). Several CRSwNP patients who fail to obtain disease control from pharmacological treatment resort to Endoscopic Sinus Surgery (ESS). Yet, it is rather common for nasal polyps to recur following nasal surgery, reaching levels between 40% and 80%, 3 to 12 years after surgery [10]. Furthermore, surgery has a good and quick effect on the immediate reduction of the size of the polyps, but it does not play any role in the reduction of type 2 inflammation and the subsequent impact on quality of life. Therefore, new treatment solutions are necessary. The recent update in treatment options with the introduction of monoclonal antibodies changed our focus on the inflammation affecting the sinonasal mucosa. The EUFOREA team released a consensus statement outlining the clinical inclusion criteria for biological treatment in CRSwNP, which underwent further refinement by the EPOS2020 expert panel [1]. Currently, three biologics are approved for use in CRSwNP: Omalizumab, Dupilumab, and Mepolizumab. While evidence suggests a higher efficacy for Dupliumab, emerging data indicate a promising contribution of Mepolizumab in improving clinical outcomes [11,12]. By preferentially binding to interleukin-5 (IL-5), Mepolizumab (SB-240563), a fully humanized IgG1/k-class monoclonal antibody, inhibits its downstream function by blocking its association with the IL-5 receptor alpha (IL-5Rα) (Figure 1). This high-affinity interaction ensures no interference of the drug with other cytokines, contributing to its optimal safety and tolerability profile [1]. IL-5 partakes in several biological functions of eosinophils, which are prime contributors in fostering airway inflammation. Mepolizumab is an immunological agent that plays a pivotal role in the treatment of patients with an eosinophilic phenotype. Initially approved in 2015 for severe eosinophilic asthma, its therapeutic applications have since broadened. In 2017, it gained approval for the treatment of eosinophilic granulomatosis with polyangiitis, followed by hypereosinophilic syndrome in 2020. Most recently, in 2021, it received FDA approval for use in CRSwNP, following the SYNAPSE trial [13,14,15]. Since Mepolizumab directly disrupts the pathogenesis of the aforementioned disorders, it is effective in treating eosinophilic allergic phenotypes. In asthma, inhaled allergens are processed by antigen-presenting cells (APCs) residing in the airway epithelium. Upon antigen recognition and activation by APCs, naïve T cell priming occurs, and they differentiate into T-helper type 2 (TH2) cells. These TH2 cells secrete cytokines, including IL-4, IL-5, and IL-13, which orchestrate the allergic inflammatory response. IL-4 and IL-13 prompt B-cell class switching to immunoglobulin E (IgE) production. IgE binds to high-affinity receptors on mast cells, sensitizing them to the specific allergen. Upon re-exposure, allergen cross-linking of bound IgE triggers mast cell degranulation, releasing mediators such as histamine and leukotrienes, leading to immediate bronchoconstriction and airway hyperresponsiveness. Mast cells also release chemotactic factors that recruit eosinophils to the airway. IL-5, predominantly produced by TH2 cells, is crucial for the proliferation, differentiation, and activation of eosinophils. Activated eosinophils release cytotoxic granules and reactive oxygen species, causing epithelial damage, mucus hypersecretion, and airway remodeling, which contribute to the chronicity and severity of asthma [16]. The majority of patients with CRSwNP exhibit a distinctive accumulation of eosinophils invading the nasal mucosa. Prolonged eosinophil survival, perhaps as a result of the defense against cell death by locally generated cytokines, such as IL-5, in the nasal tissue, may increase this buildup. Granule proteins, enzymes, cytokines, chemokines, growth factors, lipids, and oxidative products are among the numerous products that eosinophils produce and secrete after activation and that support type 2 inflammatory reactions. Several important cytokines, such as IL-5, Granulocyte-macrophage colony-stimulating factor (GM-CSF), and IL-3, control the growth and maturation of eosinophils in the bone marrow. Specifically, eosinophil differentiation, migration, induction, and survival depend on IL-5. Transmembrane α and β chains make up IL-5 receptors (IL-5R), which are responsible for IL-5 signaling. IL-5 can attach to a soluble form of the IL-5Rα chain, which inhibits IL-5R signaling [17,18]. Eosinophil activation state, maturation, and location all affect the relative expression of transmembrane and soluble IL-5Rα, which in turn affects the susceptibility of eosinophils to IL-5. According to the guidelines elaborated by the Global Initiative for Asthma (GINA), Mepolizumab has been authorized since 2015 as an additional maintenance medication for patients older than 6 years who have moderate-to-severe Eosinophilic Asthma (SEA) that does not improve with maximal conventional therapy [3,8]. More recently, the FDA (Food and Drug Administration) and EMA (European Medicines Agency) extended Mepolizumab to hypereosinophilic syndrome, Eosinophilic Granulomatosis with Polyangiitis (EGPA), and CRSwNP. For adult patients with severe CRSwNP (defined as an NPS score of ≥5 or a SNOT-22 score of ≥50) who were unable to achieve adequate disease control with OCSs and/or surgery, the Italian Agency of Drugs (AIFA) approved Mepolizumab in March 2023 as an adjunct to standard medical treatments, such as saline rinses and INCs [19]. According to the current literature, all real-life studies conducted in Italy include only studies in which the administration of biologic therapy with Mepolizumab was prescribed by pulmonologists or allergists, rather than otolaryngologists, for patients affected by severe uncontrolled CRSwNP [2,20]. The purpose of this study is to describe our experience using Mepolizumab to treat CRSwNP patients. Specifically, we evaluated the disease control and efficacy of this biologic therapy on the sinonasal aspects of severe, uncontrolled CRSwNP within an interdisciplinary care unit. 

## 2. Materials and Methods

### 2.1. Population and Study Design

We performed a single-center prospective observational study in a real-world environment at the Fondazione Policlinico Universitario Campus Bio-Medico in Rome, Italy, at the Unit of Integrated Therapies in Otolaryngology. We initially enrolled 23 consecutive patients from March to October 2023 with severe CRSwNP, treated with Mepolizumab in addition to the standard of care. Before the enrollment, each patient underwent an interdisciplinary evaluation involving, when necessary, a pulmonologist and an allergologist. Unlike other Italian real-life studies proposed in the literature, we decided not to include patients treated with Mepolizumab upon pulmonology or immunological prescription [2,20]. Following the AIFA guidelines, Mepolizumab was administered to the patients through 100 mg subcutaneous injection once every four weeks as an add-on therapy to INCS. The following criteria were applied for inclusion: (1) patients over the age of 18; (2) diagnosis of severe CRSwNP not managed by standard medications; and (3) blood eosinophil counts > 150 cells/mL, in accordance with the AIFA recommendations and the EPS/EUFOREA update [19,21]. Patients under the age of 18; pregnant women; patients who did not give their consent to begin biological therapy; patients who have undergone other biological or immunosuppressive treatment, even for different reasons; cancer patients receiving adjuvant therapy or who have received chemotherapy or radiation therapy within the last 12 months; and patients receiving concurrent long-term steroid therapy for chronic autoimmune conditions are all excluded from receiving Mepolizumab treatment. All patients provided their informed consent at the time of the initial data collection regarding the privacy and use of clinical data. The analysis of clinical data was performed anonymously. The study was conducted in accordance with the World Medical Association (WMA) Declaration of Helsinki, and ethical principles for medical research involving human participants (75th WMA General Assembly, Helsinki, Finland, October 2024). Informed consent was obtained from all subjects involved in the cohort observational study.

### 2.2. Methodology and Efficacy Outcomes

Prior to beginning Mepolizumab (T0), the patients’ baseline characteristics were evaluated. Follow-up appointments were then planned at six-month intervals: T1 at six months of treatment; T2 at twelve months. A thorough medical history was evaluated at T0, including sex, age, the existence of concurrent asthma, allergies, and prior surgery for the treatment of CRSwNP. A blood test with complete blood counts to track the eosinophil count, endoscopic evaluation, quality of life, and control of sinonasal symptoms was also performed on the patients. Finally, in order to assess sinonasal illness radiologically, each patient also needed a sinus Computed Tomography (CT) scan. At T1 and T2, the same evaluation—aside from the CT scan—was carried out.

### 2.3. Nasal Endoscopic Evaluation

To assess the dimensions of the polyps, we evaluated the patients’ Nasal Polyps Score (NPS), defined as the sum of the scores of each side of the nasal cavity [22]. The scores ranged from 0 to 4 as follows:

- 0 = no polyps;
- 1 = small polyps in the middle meatus not reaching below the inferior border of the middle turbinate;
- 2 = polyps reaching below the lower border of the middle turbinate;
- 3 = large polyps reaching the lower border of the inferior turbinate or polyps medial to the middle turbinate;
- 4 = large polyps causing complete obstruction of the inferior nasal cavity.

### 2.4. Quality of Life and Sinonasal Symptom Control

To assess the symptoms of the patients, we performed the validated Italian version of the Sinonasal Outcome Test 22 (SNOT-22). This descriptive questionnaire analyzes symptom severity, the social and emotional impact of the disease, the impact on productivity, and the sleep consequences in five domains: (1) mobility, (2) self-care, (3) usual activities, (4) pain/discomfort, and (5) anxiety/depression. Items are scored from 0 (no problem) to 5 (problem as bad as it can be) and are summed to form a total score ranging from 0 to 110. Symptomatology is considered mild at 8–20 points, moderate at 21–50 points, and severe at 51 points or above [23]. An increase of at least 8.9 points in the follow-up is considered clinically important [24].

The Visual Analogue Scale (VAS), based on a scale of values from 0 (no discomfort) to 10 (highest discomfort), assesses the severity of total symptoms [25]. The VAS was used to evaluate the level of nasal obstruction, rhinorrhea, and smell perceived by the patient. Moreover, all Adverse Events (AEs) were collected during the follow-up period every three months. The minimum follow-up period was six months [26].

### 2.5. Statistical Analysis

**Figure 2:**
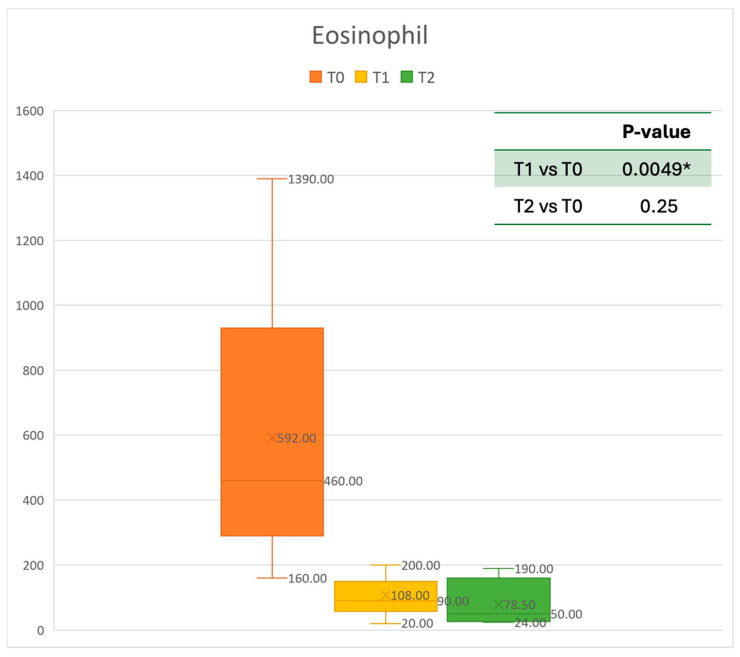
Eosinophil count at baseline (T0), after six months of treatment (T1), and 12 months of treatment (T2); * denotes a statistically significant result (*p* < 0.05).

**Figure 3:**
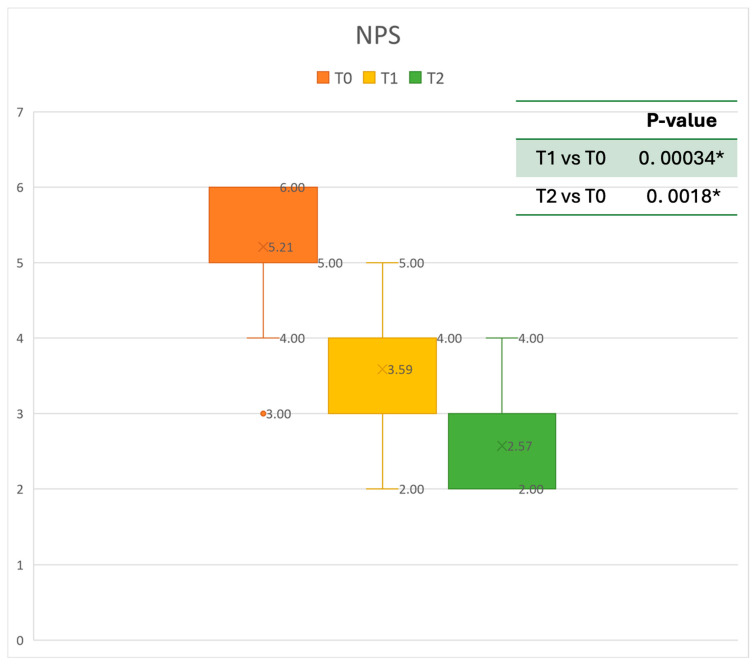
Nasal Polyps Score (NPS) at baseline (T0), after six months of treatment (T1), and 12 months of treatment (T2); * denotes a statistically significant result (*p* < 0.05).

**Figure 4:**
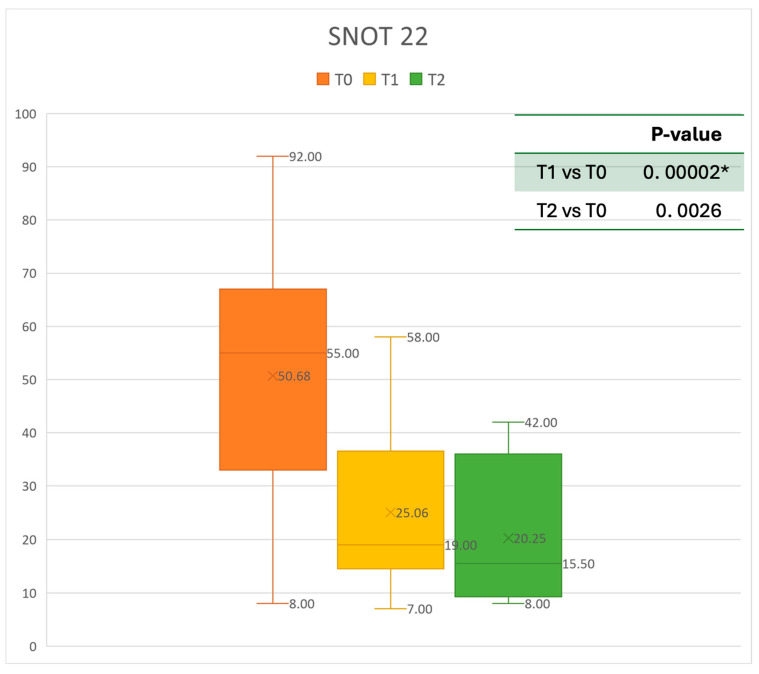
SNOT 22 at baseline (T0), after six months of treatment (T1), and 12 months of treatment (T2); * denotes a statistically significant result (*p* < 0.05).

**Figure 5:**
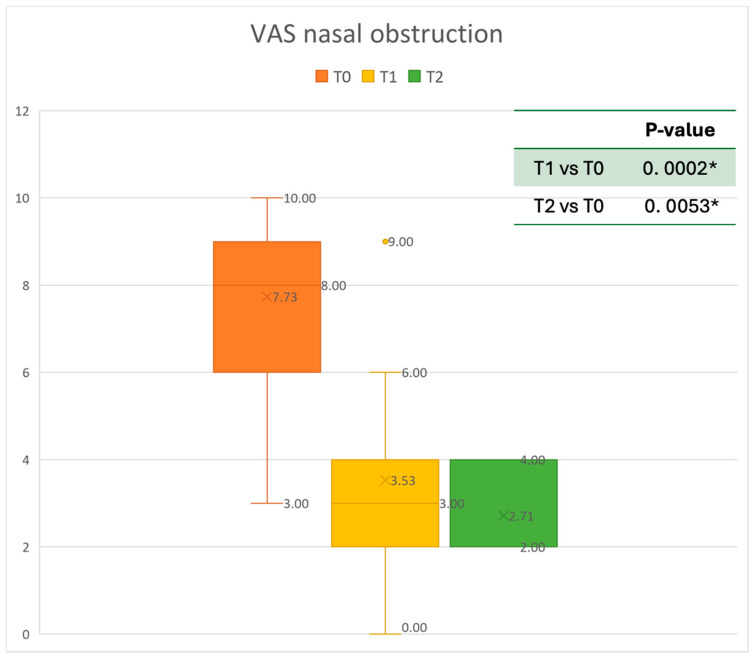
VAS of nasal obstruction at baseline (T0), after six months of treatment (T1), and 12 months of treatment (T2); * denotes a statistically significant result (*p* < 0.05).

**Figure 6:**
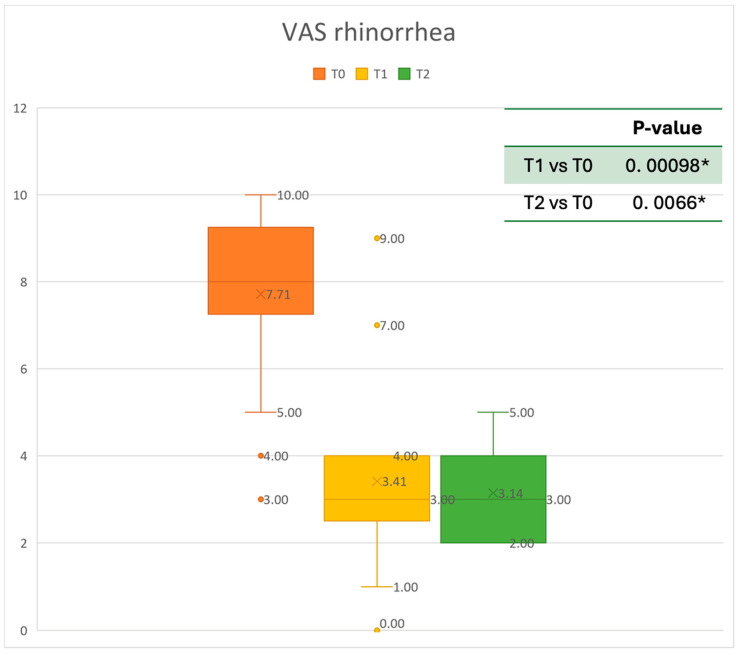
VAS of rhinorrhea symptoms at baseline (T0), after six months of treatment (T1), and 12 months of treatment (T2); * denotes a statistically significant result (*p* < 0.05).

**Figure 7:**
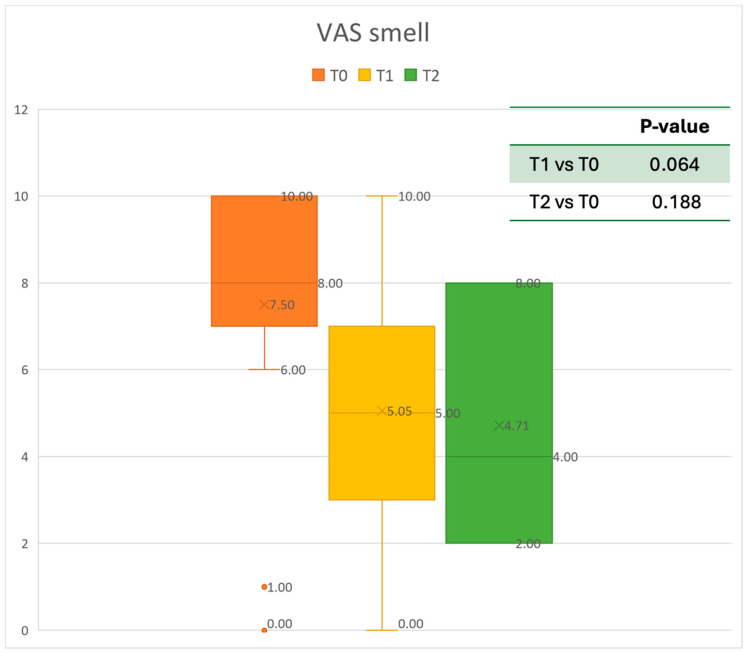
VAS of smell perception at baseline (T0), after six months of treatment (T1), and 12 months of treatment (T2).

Data were analyzed using MATLAB R2022b ^®^ by MathWorks. Results are presented as mean ± standard deviation. The normality of the data was assessed using the Anderson–Darling test (adtest function). A paired *t*-test was utilized for normally distributed data (*t*-test function) and Wilcoxon’s signed-rank test was used to investigate matched pairs of non-normally distributed data (signrank function) in order to compare the results at 6 and 12 months after Mepolizumab delivery. A *p*-value of <0.05 was considered statistically significant. Figure 2, Figure 3, Figure 4, Figure 5, Figure 6 and Figure 7 were generated using Microsoft Excel to visualize and present the data.

## 3. Results

At the end of our selection process, we included 20 severe CRSwNP patients treated with Mepolizumab for 12 months. Of these, 14 were males, and 6 were females, showing a male prevalence. Three patients discontinued the study: one due to pregnancy and two others because they stopped treatment before completing six months. The mean age was 57.63 ± 16.99 years. Nine patients had concomitant asthma (not severe). Other comorbidities mainly included Obstructive Sleep Apnea (n = 2), Chronic Obstructive Pulmonary Disease (n = 2), ischemic heart disease (n = 2), hypercholesterolemia (n = 1), serous otitis media (n = 1), insulin resistance (n = 1), and glaucoma (n = 1). Moreover, two patients also showed Nonsteroidal anti-inflammatory drug (NSAID)-exacerbated respiratory disease (NERD)-induced urticaria/angioedema/anaphylaxis. None of the included patients had a history of atopic dermatitis. The total mean was 1.5 surgeries per subject (ranging from 1.0 to 4.0 surgeries). Among the included patients, one had never undergone ESS for CRSwNP because he was deemed unfit for surgery (previous sudden cardiac arrest and severe coronary artery disease), and sixteen patients underwent at least one ESS before starting Mepolizumab. Eleven patients (64.7%) had allergies, with just one being to perennial allergens (Dermatophagoides Pteronissunus). The remaining patients were allergic to grasses, cypress, and *Parietaria officinalis*. Mepolizumab appeared to be well tolerated by all patients. Throughout the duration of the investigation, INCS and nasal saline irrigations were administered to all participants. Specifically, each participant administered 100 mcg of mometasone furoate twice daily into each nostril. Prednisone usage and exacerbations—defined as episodes requiring intramuscular injection or oral corticosteroid therapy for at least 3 days—were documented at every stage of the study. Prior to Mepolizumab, there were 20 patients who were reliant on OCSs; following 6 and 12 months of treatment, the number dropped to 4 and 2, respectively. Both of these patients (100%) had asthma. Among the AEs registered, one patient developed severe rhinopharyngitis after the first administration. Moreover, two of the nine patients with asthma complained of exacerbations. In two cases, a short course of OCSs was prescribed twice as a salvage therapy due to worsening clinical symptoms. No patient required ESS during the treatment period.

The demographic characteristics of the patients are reported in Table 1.

### 3.1. Eosinophilic Count Trend

Concerning blood tests, eosinophils decreased gradually. The mean absolute value of 0.592 cells × 10^9^/L before treatment decreased to 0.108 cells × 10^9^/L at T1. This resulted in a mean gain of 0.473 cells × 10^9^/L after 6 months, corresponding to a 68% improvement. After one year, the mean number of eosinophils decreased to 0.0785 cells × 10^9^/L. A statistically significant improvement was seen between T0 and T1. The results are reported in Figure 2.

### 3.2. Nasal Endoscopic Evaluation

Regarding the NPS, the mean value before starting Mepolizumab was 5.21 ± 0.79. After six months of treatment, the score improved to 3.59 ± 0.87, and after 12 months, there was further improvement, reaching a mean value of 2.57 ± 0.79. After 6 months, an improvement of 30% was observed, corresponding to a loss of 1.59 points. A statistically significant improvement was seen between T0 and T1 and T0 and T2. The results are reported in Figure 3.

### 3.3. Quality of Life and Sinonasal Symptom Control

The mean SNOT-22 value at baseline was 50.68 ± 23.32; after six months, the value decreased to 25.06 ± 15.62, and after twelve months it was 20.25 ± 15.06. After 6 months, an improvement of 50% was observed, corresponding to a loss of 29.82 points. A statistically significant improvement was seen between T0 and T1 and T0 and T2. The results are reported in Figure 4. The nasal obstruction VAS decreased from 7.71 ± 2.02 at baseline to 3.22 ± 1.52 at T1 (improvement of 57%) and 2.71 ± 0.95 at T2 (Figure 5). The rhinorrhea VAS decreased from 7.71 ± 2.20 at baseline to 3.41 ± 2.06 at T1 (improvement of 47%) and 3.14 ± 1.21 at T2 (Figure 6). The smell loss VAS decreased from 7.47 ± 3.11 at baseline to 4.83 ± 2.81 at T1 (improvement of 36%) and 4.71 ± 2.63 at T2. While nasal obstruction and rhinorrhea decreased significantly from baseline to T1 and T2, smell did not improve from the baseline (Figure 7). All the results are summarized in Figure 3.

## 4. Discussion

Biologic therapies represent a valid alternative in patients with severe CRSwNP. Several monoclonal antibodies (mAbs) have been recently approved for treating a number of type 2 immune response-associated conditions, including CRSwNP and severe asthma, displaying minimal side effects [27]. Mepolizumab, a humanized monoclonal antibody, targets IL-5, a cytokine which fosters eosinophil recruitment, maturation, and survival. In particular, Mepolizumab prevents its engagement with IL-5Rα, while apparently sparing other cytokine pathways due to its high-affinity interaction, with an optimal safety and tolerance profile [28]. Mepolizumab has received worldwide approval for the treatment of severe eosinophilic asthma and CRSwNP at a dose of 100 mg, administered subcutaneously, at a dose of 300 mg for patients with EGPA and hypereosinophilic syndrome [29]. The SYNAPSE study is a randomized, double-blind, placebo-controlled, parallel-group phase 3 study exploring the use of Mepolizumab in 407 patients affected by severe CRSwNP (206 patients were allocated to the experimental group, 201 to the control arm). In this study, Mepolizumab also appeared to reduce the actual number of sinus surgeries and the use of OCSs and to improve sinonasal symptoms, while displaying an acceptable safety profile [13]. According to the first real-life study conducted by Domínguez-Sosa MS and colleagues [16], Mepolizumab significantly relieved clinical symptoms, blood eosinophils, polyp scores, and the use of OCSs at the 1 year follow-up, resulting in an increased quality of life in 55 subjects affected by severe CRSwNP, regardless of the presence or absence of asthma or NERD. To our knowledge, this is the first study conducted in real-life settings aimed at evaluating the impact of Mepolizumab on severe CRSwNP patients in Italy. Unlike other real-life trials already present in the literature, we excluded patients receiving Mepolizumab via pulmonological or immunological prescription [2,20]. The results of our observational study validate that 100 mg of Mepolizumab, given subcutaneously at home using a pre-filled auto-injector every four weeks (per the Italian Medicines Agency’s guidelines), is an effective treatment for severe, uncontrolled CRSwNP. Mepolizumab administration resulted in a notable improvement in all primary outcomes, including the NPS score, SNOT-22 score, VAS obstruction, and rhinorrhea. This trend was observed during all 12 months of treatment. However, our 6-month long analysis, in the six months revealed a mismatch between NPS and SNOT-22 improvements (30% vs. 50%, respectively), confirming that the improvement of nasal symptoms does not necessarily reflect the reduction in polyp dimensions. Regarding smell loss, the VAS dropped from 7.47 ± 3.11 at baseline to 4.71 ± 2.63 at T2, without reaching statistical significance. However, this reduction may still hold clinical significance, as improvements in smell function, even if modest, can have a meaningful impact on patients’ quality of life. It is also worth noting that the lack of statistical significance may be influenced not only by the small sample size but also by the limited follow-up duration, which may have constrained the detection of further changes over time. In addition, a significant reduction in baseline T0 blood eosinophils 0.592 cells × 10^9^/L to post-treatment (T6) blood eosinophils 0.108 cells × 10^9^/L, (*p* < 0.001) and (T12) blood eosinophils 0.079 cells × 10^9^/L was noted with the administration of Mepolizumab. Headaches, epistaxis, sinusitis, and nasopharyngitis were the most frequently reported adverse effects in the literature. In this real-life study, our observations confirmed the same safety profile of Mepolizumab as previously described in other articles, with one single case of nasopharyngitis being reported. To better define the impact and frequency of these AEs [13], studies with a long-term follow-up are needed. Identifying inflammatory phenotypes and distinguishing between type 2 and non-type 2 inflammation, is pivotal for determining eligibility for targeted biologic therapies in both asthma and comorbid CRSwNP settings. In this study, each patient underwent a thorough interdisciplinary assessment involving an ENT specialist, an immunologist, and a pulmonologist. This approach allowed a comprehensive assessment of symptoms and clinical history, ensured an accurate clinical diagnosis, and guided the identification of the best treatment strategy to manage disease progression and prevent exacerbations. Importantly, this approach can identify patients who may benefit from biologics in the earliest stages of disease [30]. Currently, an indication for biological treatment includes patients who previously underwent ESS but are excluded due to exceptional circumstances (e.g., not fit for surgery). In our real-life study, we included only one patient who never underwent ESS for previous sudden cardiac arrest and severe coronary artery disease. Moreover, it was debated whether the extent of the surgery needs to be evaluated before considering the choice of a biologic. However, because we do not have sufficient data proving that more extensive surgery results in better outcomes in patients with CRSwNP, this criterion was not added [21]. Moreover, these strategies are not mutually exclusive and should be regarded as complementary to maximize therapeutic outcomes and to choose the most effective, minimally invasive strategy. While ESS has consistently been shown to improve polyp burden faster than biological therapy, ESS does not treat the inflammatory response in CRSwNP patients. Studies suggest that biologics produce a systemic reduction in type 2 inflammation and the subsequent impact on subjective quality of life [31]. No comparative head-to-head studies among different biologics are available. Comparing our findings with previous real-life studies in the literature, Cavaliere et al. [20] displayed similar results, showing a significant improvement in NPS, quality of life, and nasal symptoms in 20 patients affected by CRSwNP. Nevertheless, in contrast to our study, these patients’ loss of smell VAS was significantly improved. It is crucial to emphasize, nonetheless, that the authors also included patients with asthma and CRSwNP who were receiving Mepolizumab via a pneumological and immunological prescription. Likewise, following 12 months of Mepolizumab treatment, Galletti and colleagues [32] found a statistically significant improvement in SNOT-22 and NPS in the 6th and 12th months, compared to baseline values in 67 patients. Also in this instance, the loss of smell significantly improved, which was something we did not observe in our investigation. However, this is most likely due to the small number of patients included in this study and to a limited follow-up. The choice of the biologic to be used in the treatment of CRSwNP depends on the good phenotyping and endotyping of the patient; therefore, by refining the patient selection, we would have the best results in terms of efficacy and safety. Multiple potential limiting factors should be taken into account when interpreting our findings. First, the sample size is relatively small. Second, being an observational study, all participants received Mepolizumab and there was no control arm. It is important to mention that while the VAS is a valid tool for symptomatic assessment, it is inherently subjective and may be influenced by individual perception (including patient expectations, mood, or cognitive biases) and external factors, such as treatment interactions. Likewise, we analyzed a relatively small number of clinical variables, due to the complexity of the disease, which requires multifactorial considerations, while focusing on the variables commonly validated in the existing literature to ensure consistency and comparability. Additionally, participants did not undergo any imaging assessments to evaluate changes following the Mepolizumab course, as post-treatment CT scans were avoided to reduce unnecessary radiation exposure. Moreover, such imaging assessments are only used for refractory patients awaiting further surgery or when clinical complications are suspected. Finally, the follow-up period was limited to one year of treatment. Further research is necessary to confirm these findings, better explore the underlying immune pathophysiology, and identify future research directions.

## 5. Conclusions

Our preliminary findings highlight the efficacy of Mepolizumab in the therapeutic approach for patients with CRSwNP. The treatment has been associated with a reduction in nasal polyps, a decrease in blood eosinophils, the improvement of disease-related symptoms, and an ameliorated QoL. However, further large-scale studies with an extended follow-up are needed to validate these promising outcomes.

## Figures and Tables

**Table 1:** Demographic characteristics of the included patients.

Number of patients	20
Sex (M/F)	14/6
Age (year)	57.63 ± 16.99
Allergy to inhalants (Y/N)	7/13
Allergy to ASA (Y/N)	2/18
Asma (Y/N)	8/12
Number of previous FESS	0 FESS: 1 1 FESS: 10 2 FESS: 3 3 FESS: 2 4 FESS: 1

## Data Availability

The data supporting this study’s findings are not openly available due to reasons of sensitivity and are available from the corresponding author upon reasonable request.
